# Some paradoxes and unresolved aspects of hepatic de novo lipogenesis

**DOI:** 10.1038/s44324-024-00020-7

**Published:** 2024-08-02

**Authors:** John G. Jones

**Affiliations:** Center for Neurosciences and Cell Biology, University of Coimbra, Coimbra, Portugal

**Keywords:** Biochemistry, Physiology

## Abstract

Hepatic de novo lipogenesis (DNL) is a critical pathway in both liver intermediary metabolism and whole-body nutrient management. In the setting of excessive caloric intake, increased DNL fluxes are implicated in the pathogenesis of metabolic-associated steatotic liver disease (MASLD). As a result, there is intense interest both in the measurement of DNL activity and in gaining a better understanding on how this drives MASLD development. While much progress has been made towards these objectives, a number of intriguing uncertainties and paradoxes remain. This short perspective will focus on some of these aspects, namely a), how DNL contributes to triglyceride overload, b), the timing of DNL pathway activation with nutrient availability, c) the sources of acetyl-CoA for DNL and d), the sources of NADPH reducing equivalents for DNL. The implications of these uncertainties on pharmacological targeting of hepatic DNL activity will also be discussed.

## Introduction

The biosynthesis of fatty acids from acetyl-CoA—a process known as de novo lipogenesis (DNL)—is a critical component of nutrient conversion to lipid for long-term storage. The liver is among the first organs in the body to receive absorbed nutrients and is the principal site for their initial metabolism. Hepatic metabolism of lipid and carbohydrate under these fed conditions, as well as in the fasted state, is a cornerstone of whole-body fuel substrate management—most notably ensuring near-constant levels of circulating glucose as well as efficient storage of excess nutrient as lipid. While the liver is not the only tissue in the body with the capacity for DNL, it is by far the most active in terms of DNL fluxes per gram of tissue and also the most dynamic in terms of DNL flux modulation.

In the healthy state, liver triglyceride levels are 1–2% by weight. Metabolic-associated steatotic liver disease (MASLD) is defined as liver triglyceride concentrations exceeding 5.5%^1^, i.e. a 2 to 4-fold increase over healthy levels. While this might seem at first glance to be a substantial difference, in terms of total body lipid it is an insignificant amount. Assuming a 1.5 kg liver mass, a gain in liver triglyceride levels from 1% to 6% represents an increase of 1.5 × 0.05 = 75 grams of lipid—an amount that would be essentially undetectable if it was incorporated instead into adipose tissue. Conversely, when subjects with MASLD are placed on a restricted caloric diet decreases in hepatic triglyceride levels are observed well in advance of any significant drops in either total body weight or fat mass^2^. Therefore, while significant alterations in the hepatic triglyceride pool size barely register in terms of total whole-body triglyceride levels, they can have profound effects on hepatic health and function.

## How does DNL enable hepatic triglyceride accumulation?

Hepatic triglyceride levels are normally kept in balance through the matching of fatty acid appearance rates with those of fatty acid oxidation plus export of triglyceride via very-low-density lipoprotein. Hepatic DNL activity is now considered to be a key determinant of increased hepatic fatty acid appearance during MASLD development^3–6^. Hepatic triglyceride is formed by the sequential esterification of glycerol-3-phosphate by fatty acyl-CoA with an estimated 15–25% of these originating from DNL^6^. Depending on the prandial state, the majority of fatty acid precursors originate either from dietary lipid via chylomicron remnants, or from circulating free-fatty acids released by adipose tissue lipolysis. These fatty acids include both essential and non-essential species with each of the three esterification enzymes having different affinities for saturated, monounsaturated and polyunsaturated fatty acids. DNL (which in this context also includes fatty acid elongation and desaturation) contributes either saturated or monounsaturated fatty acids to this pool. Specific fatty acyl-CoA precursors are also recruited for the biosynthesis of other lipid species, notably palmitoyl-CoA for ceramide formation^7^. While DNL activity directly adds to hepatic fatty acid overflow, it can also indirectly contribute via the inhibition of fatty acid β-oxidation by its malonyl-CoA intermediate. The fractional contribution of DNL to the total hepatic fatty acid pool is relatively small, while the capacity for hepatic β-oxidation is up to 45–90 g of fatty acids per day in humans. This estimate is based on hepatic Krebs cycle fluxes of 0.5–1.0 mmol/min per 1.5 kg liver mass measured by stable-isotope tracers^8,9^ and assuming that all acetyl-CoA is derived from β-oxidation of fatty acids. This rate of acetyl-CoA utilization is equivalent to 0.125–0.250 mmol/min of palmitate consumption. Over 24 h, this amounts to 180–360 mmol, or ~45–90 g of fatty acids. Ketogenesis accounts for up to an additional 10–20% of fatty acid disappearance. it is possible that the inhibitory effect of DNL on β-oxidation may be at least as important as its direct contribution of newly synthesised fatty acids. The extent to which the direct versus indirect actions of DNL contribute to hepatic triglyceride overload may depend on several factors including dietary sugar intake^10^ – notably fructose^11,12^ and the feeding/fasting state^13^. On the one hand, a number of studies have demonstrated that NAFLD onset is characterized by either *increases* in fatty acid β-oxidation^8,14,15^ or no change^9^, which appears to be inconsistent with the expected inhibitory effects of malonyl-CoA. On the other hand, the discovery of low but persistent fasting DNL activity in NAFLD subjects that showed a strong negative correlation with hepatic β-oxidation and ketogenesis^16^ demonstrates the importance of DNL in the inhibition of oxidative fatty acid clearance under these fasting conditions. Given that there is a substantial inflow of fatty acids into the liver from adipose tissue lipolysis in fasting as well as fed states^17,18^, this action is likely to be a significant contributor to elevated hepatic triglyceride levels. Finally, in the setting of chronic overfeeding and diet-induced obesity, reciprocal control of hepatic DNL and fatty acid oxidation is additionally perturbed by circadian enhancers such as Rev-erbα. These not only potentiate hepatic DNL in a periodic manner, but also unexpectedly increase hepatic fatty acid oxidation^19^.

## DNL activation and substrate availability

DNL is a metabolically expensive pathway requiring high inputs of energy and NADPH-reducing equivalents. It is therefore not surprising that DNL fluxes are controlled such that they are most active when there is a surplus of nutrients. DNL is primarily regulated at the transcriptional level through the actions of two transcription factors, carbohydrate-response element binding protein (ChREBP)^20^ and the family of sterol-response element binding proteins-1 (SREBP1) of which the SREBP-1c isoform is considered to be the most important. While ChREBP is activated by glucose-6-P and/or downstream sugar phosphates^21,22^ originating from either glucose or fructose^23^, SREBP-1c is activated by insulin and also mediates the activation of glucokinase by insulin^24,25^ while SREBP1 expression and actions are additionally modulated by circadian enhancers^19,26,27^. Thus, both SREBP-1c and ChREBP synergistically promote the conversion of glucose and fructose to lipid^28^ while circadian control of SREBP expression and other regulatory factors that mediate lipid metabolism tunes this activity to match the habitual feeding period^29^. Both SREBP-1c and ChREBP activate the transcription of key DNL pathway enzymes such as acetyl-CoA carboxylase (ACC) and fatty acid synthase (FAS)^28,30^. The combined transcription and translation of these complex proteins is estimated to take at least 10 min while hepatic uptake and metabolism of glucose (or fructose) occurs in about 1 min^31^. Other studies have demonstrated a notable lag between the intake of food and hepatic DNL activity. In cultured rat hepatocytes that had been isolated at different intervals after feeding, maximal DNL rates were found in those cells taken 5 h after feeding while the lowest rates were found midway during the feeding period^32^. In healthy males, DNL rates were found to peak at 4.2 h post-feeding^13^. It seems that by the time the DNL pathway becomes fully operational, much of the dietary sugar will have already been metabolized to either glycogen, glycolytic products or short-chain fatty acids. Moreover, there is evidence that during the initial stages of feeding, glucose or fructose conversion to glycogen via direct and/or indirect pathways is prioritized over their metabolism to lipid^33^. Finally, in the case of fructose, there is evidence that a significant fraction is metabolized to glucose and short-chain fatty acids in the intestine^34^. How then is glucose and fructose metabolism coupled with DNL?

**Fig. 1:**
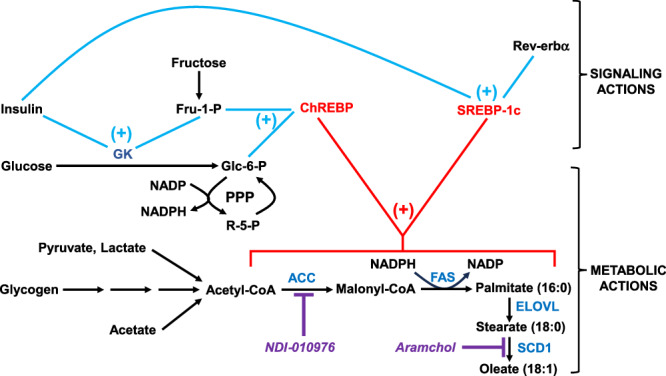
Model connecting hepatic glucose and fructose metabolism to de novo lipogenesis. In the signaling phase characterized by high sugar and insulin levels and additionally cued by circadian factors, fructose and glucose are metabolized relatively quickly to sugar phosphates—the latter via activation of glucokinase (GK) while the transcription factors ChREBP and SREBP-1c are also activated. They in turn initiate the expression of acetyl-CoA carboxylase (ACC) and fatty acid synthase (FAS) of the de novo lipogenesis pathway. By the time that this pathway is functional, a substantial proportion of ingested glucose and fructose will have already been metabolized to glycogen, pyruvate/lactate, and acetate. Thus, the metabolic flux of acetyl-CoA into de novo lipogenesis is supported by all of these precursors. Also shown are the sites of DNL pathway inhibition by NDI-010976 and Aramchol—candidate drugs for the treatment of MASLD and MASH.

Figure 1 proposes some possible mechanisms by which this might be accomplished. The appearance and initial metabolism of glucose and/or fructose to sugar phosphates coupled with high insulin levels and circadian cues activate ChREBP and SREBP-1c that in turn switch on the transcription and translation of DNL pathway enzymes. Once DNL is functional, it will utilize any available source of acetyl-CoA, including microbially-generated acetate, for fatty acid synthesis and this activity will persist until nutrient levels are depleted and the pathway is deactivated. This paradigm explains why on the one hand hepatic fatty acids can be efficiently labeled from acetate tracers regardless of prandial state and dietary sugar composition^13,35^, while on the other hand, very high intakes of glucose and fructose contribute a minority of carbons to the lipogenic acetyl-CoA pool^36,37^. Another mechanism that can account for the delay between sugar catabolism and DNL activation is that the sugars are initially metabolized to hepatic glycogen via direct and indirect pathways and subsequently become available for DNL during the initial stages of glycogenolysis. Such a mechanism is not only consistent with the observed efficient uptake of both dietary glucose and fructose into hepatic glycogen^38^, but also with the prioritization of glycogenic over lipogenic metabolism of dietary sugar in the initial feeding phase^33^. It is also interesting that DNL activity is promoted when carbohydrate flux into glycogen is attenuated from the top down, either through the physical constraints of glycogen accumulation or by defective glycogen synthase function^39^. Finally, feeding/fasting regimes that are out of phase with normal circadian rhythms can also augment DNL activity^40^. In humans undergoing a normal daily meal and sleep cycle, hepatic glycogen levels accumulate stepwise over breakfast, lunch and dinner peaking by bedtime and then falling steadily during sleep to a nadir before breakfast the following day^41^. To the extent that high hepatic glycogen levels direct dietary carbohydrate flow away from glycogenesis and towards DNL, any intake during this early night period—for example a snack taken during a night-shift—is likely to augment DNL to a greater extent compared to an equivalent intake taken at breakfast.

From a metabolic tracer perspective, investigating the possibility of glycogen as an intermediate of sugar lipogenesis is challenging because of the difficulty in tagging the relatively small and short-lived fraction of the total glycogen pool that is destined for DNL. The advent of real-time in vivo^42^ measurement of hepatic glycogen content and turnover^43,44^ coupled with sensitive and dynamic measurements of VLDL fatty acid tracer enrichment during feeding^45,46^, may provide a means of detecting and quantifying such a flux in human subjects.

## Sources of NADPH for DNL

The availability of glucose or fructose as lipogenic substrates is also pertinent to the sources of NADPH for DNL given that the formation of a single palmitate molecule requires 14 equivalents of cytosolic NADPH. If glucose or fructose are assumed to be the sole substrates for DNL, and the PPP is assumed to be the sole source of NADPH, then for every mol of hexose that is converted to palmitate, 0.23 mol need to be oxidized by the pentose phosphate pathway (PPP)^36^. If glucose is accompanied by other substrates such as lactate or acetate, then fraction of glucose-6-P oxidized via the PPP *versus* that incorporated into lipid needs to be increased. In mice fed diets supplemented with high levels of glucose and fructose, the total contributions of these sugars to the acetyl-CoA precursor pool of DNL was around 50%, implying that the mol fraction of glucose-6-P oxidized by the PPP relative to that incorporated into fatty acids would need to be ~0.40. In an earlier study of mice fed a diet supplemented with similar amounts of glucose and fructose, glucose incorporation into fatty acid was tracked with [U-^13^C]glucose. At the same time, glucose metabolism by the PPP that was directly linked to DNL was assessed by measuring the transfer of [3-^2^H] and [U-^2^H_7_]glucose deuterium into fatty acids^47^. These studies revealed that for each mol of glucose incorporated into fatty acids, approximately 0.46 moles were utilized by lipogenic PPP activity. However, when the fraction of glucose-6-P metabolized by the PPP was measured by glycogen isotopomer analysis following enrichment from [U-^13^C]glucose or [U-^13^C]fructose, the estimate was much smaller (0.10–0.14)^36,47^. These discrepancies can be reconciled by the fact that total glucose-6-P flux is likely to be far in excess of glucose-6-P recruited for DNL. Thus, in terms of absolute fluxes, a 10-14% utilization of total glucose-6-P by the PPP can include a 46% PPP utilization of glucose-6-P destined for DNL. Therefore, contrary to our previous conclusions^36^, it appears that there is indeed a sufficient level of glucose-6-P oxidation via the PPP to sustain the lipogenic incorporation of approximately one acetyl-CoA from non-sugar substrates for each one contributed by glucose-6-P. These studies also support the postulate that while DNL may recruit a substantial proportion of acetyl-CoA from non-sugar precursors, it is nevertheless coupled to glucose-6-P production because of the requirements for NADPH production via the PPP. However, alternative pathways of NADPH production exist in the liver that do not directly rely on glucose-6-P including cytosolic NADP-isocitrate dehydrogenase^48^, serine oxidation to glycine via the one-carbon pathway^49^, and transfer of reducing hydrogen from NADP to NADPH via nicotinamide nucleotide transhydrogenase^50^. To the extent that NADPH from these pathways is used by DNL, its dependence on glucose-6-P availability is decreased.

## Pharmacological targeting of DNL

The disproportionate impact of DNL on total hepatic lipid levels and the non-essential nature of DNL products make it a highly attractive target for pharmacological inhibition. Currently, there are a growing number of investigational drugs that inhibit specific enzymes of the DNL pathway. As such, they act downstream of DNL activation and substrate availability and are therefore insensitive to these parameters. Aramchol, a fatty acid-bile acid conjugate that is a partial inhibitor of stearoyl-CoA dehydrogenase-1 (SCD-1), was shown to significantly decrease liver triglycerides in a cohort of subjects with MASLD^51^. In another study of MASH patients treated with various doses of aramchol, there were no significant alterations in liver triglyceride levels but there were improvements in both liver histology and circulating transaminases^52^. Both studies also reported good tolerance and low side effects from this drug. Inhibition of ACC is of particular interest since not only does this block DNL but also removes the malonyl-CoA brake on mitochondrial long-chain fatty acid oxidation. NDI‐010976, an allosteric inhibitor of ACC1 and ACC2, was administered to overweight healthy subjects who had also ingested fructose in order to stimulate hepatic DNL, which was measured by analysis of VLDL-palmitate ^13^C-enrichment following infusion with [1-^13^C]acetate^53^. NDI‐010976 caused a significant dose-dependent reduction of hepatic DNL and was also well tolerated by the subjects.
